# Neuronal oxidative damage and dendritic degeneration following activation of CD14-dependent innate immune response *in vivo*

**DOI:** 10.1186/1742-2094-1-20

**Published:** 2004-10-21

**Authors:** Dejan Milatovic, Snjezana Zaja-Milatovic, Kathleen S Montine, Feng-Shiun Shie, Thomas J Montine

**Affiliations:** 1 Department of Pathology, University of Washington, Harborview Medical Center, Seattle Washington 98104, USA

## Abstract

The cause-and-effect relationship between innate immune activation and neurodegeneration has been difficult to prove in complex animal models and patients. Here we review findings from a model of direct innate immune activation via CD14 stimulation using intracerebroventricular injection of lipopolysaccharide. These data show that CD14-dependent innate immune activation in cerebrum leads to the closely linked outcomes of neuronal membrane oxidative damage and dendritic degeneration. Both forms of neuronal damage could be blocked by ibuprofen and alpha-tocopherol, but not naproxen or gamma-tocopherol, at pharmacologically relevant concentrations. This model provides a convenient method to determine effective agents and their appropriate dose ranges for protecting neurons from CD14-activated innate immunity-mediated damage, and can guide drug development for diseases, such as Alzheimer disease, that are thought to derive in part from CD14-activated innate immune response.

## Introduction

Activated innate immunity is associated with several degenerative and destructive brain diseases including Alzheimer disease (AD), HIV-associated dementia (HAD), ischemia, head trauma, stroke, cerebral palsy, and axonal degeneration in multiple sclerosis [1]. In this complex response, some aspects are proposed to be neurotrophic, others neurotoxic, and each potentially a consequence rather than a contributor to neurodegeneration. Indeed, a severe limitation to understanding the precise role of innate immunity in these diseases and their corresponding animal models is that innate immunity is activated simultaneously with multiple other stressors and responses to injury, thereby greatly confounding any clear conclusion about cause-and-effect relationships. For these reasons we have adopted a simple but highly specific model of isolated innate immune activation: intracerebroventricular (ICV) injection of low dose lipopolysaccharide (LPS).

LPS specifically activates innate immunity in peripheral organs through a well-described Toll-like receptor (TLR)-dependent signaling pathway [2,3]. There are 9 known human plasma membrane-spanning TLRs expressed in many cell types throughout the body that have been discovered in the context of innate immune response to micro-organisms. TLR-mediated innate immune response can be considered in three phases: initial signal transduction cascade, secondary signaling cascades, and effectors. The initial signaling cascade starts with ligand activating one of the 9 plasma membrane TLRs. All of these receptors require the adaptor protein MyD88 for immediate response to LPS and initiate a bifurcated signal transduction cascade that culminates in altered gene transcription, primarily via NF-κB activation but also through c-Fos/c-Jun-dependent pathways. Some of the activated gene transcripts encode directly for receptor ligands while others are enzymes that catalyze the formation of receptor ligands that in turn activate secondary autocrine and paracrine signaling cascades. These signaling events culminate in the generation of effector molecules including bacteriocidal molecules, primarily free radicals generated by NADPH oxidase and myeloperoxidase (MPO), as well as cytokines and chemokines that can attract an adaptive immune response. Although originally identified as part of the response to exogenous antigens from micro-organisms, a broader pathophysiologic role for TLR-dependent signaling in response to endogenous ligands in now clear. Indeed, from this perspective, the effectors at the culmination of these signaling pathways are more appropriately viewed as cytocidal rather than specifically bacteriocidal. The precise agents responsible for cytocidal activity are not clearly established but likely include free radicals generated principally by NADPH oxidase, MPO, and inducible nitric oxide synthase (iNOS) in combination with cytokines and chemokines.

TLR-4 is the receptor for LPS in peripheral organs [2,3]. However, another protein, CD14 is critical to LPS activation of TLR-4. Membrane-anchored CD14 is now thought to act a co-receptor for LPS but not to initiate intracellular signaling cascades. It is important to note that CD14 serves a similar function with TLR-2, although the activating agents here are bacterial products other than LPS [4]. Within minutes to hours of exposure to LPS, there is increased gene transcription and subsequent translation of cytokines and chemokines, prominently including tumor necrosis factor, interleukin-1, and interferons, as well as several enzymes; important among these are iNOS and cyclooxygenase 2 (COX-2) that catalyze the formation of NO and prostaglandin (PG) H_2_, respectively [4]. While NO is a potent cell signaling molecule, PGH_2 _has relatively low receptor binding affinity but is rapidly and efficiently converted to multiple PGs or thromboxane A2, each of which are potent activators of a large family of G protein-coupled receptors [5]. The combination of these initial and secondary signaling cascades produces a robust innate immune response. This same response can occur in response to endogenous ligands that also activate the CD14/TLR-4 pathway [2,3]. Indeed, several endogenous CD14/TLR ligands have received increasing attention for their potential roles in human diseases [6], and polymorphisms in TLR-4 are associated with risk for atherosclerosis and asthma, as well as other human diseases [7]. With respect to AD, amyloid beta (A) fibrils have been shown to activate the microglial innate immune response through CD14-dependent mechanisms [8]. Relevant to a broader range of neurodegenerative diseases, novel peptides and neoantigens exposed by apoptotic cells [9] also activate CD14-dependent innate immune response in macrophages. While none of these data point to CD14 or innate immune response as etiological in neurodegenerative disorders, these findings from *in vitro *and cell culture experiments raise the possibility that CD14-dependent signaling may be a common process shared in the pathogenesis of neurodegenerative diseases, especially AD.

Here we present our results from studies that have identified the molecular and pharmacologic determinants of ICV LPS-initiated cerebral neuronal damage *in vivo*. It is important to stress that several laboratories have shown that glia, predominantly microglia, are activated by LPS but that neurons do not respond to LPS because they lack the appropriate receptors [10,11]. We measured two main endpoints; one biochemical and one structural. Since free radicals are a primary mechanism of cytocidal activity from innate immune response, we used a stable isotope dilution method with gas chromatography and negative ion chemical ionization mass spectrometry to quantify compounds formed by free radical attack on the neuronal membrane-enriched fatty acid, docosohexaenoic acid (DHA); we have termed these molecules F_4_-neuroprostanes (F_4_-NeuroPs) [12]. In addition to this biochemical marker of neuronal oxidative damage, we directly quantified neuron number as well as dendrite length and spine density in pyramidal neurons of hippocampal sector CA1 using the Golgi impregnation technique followed by quantitative morphometry with Neurolucida (MicroBrightField, VT) [13].

### Lack of adaptive immune response, fever, or structural damage to brain following ICV LPS

**Figure 1:**
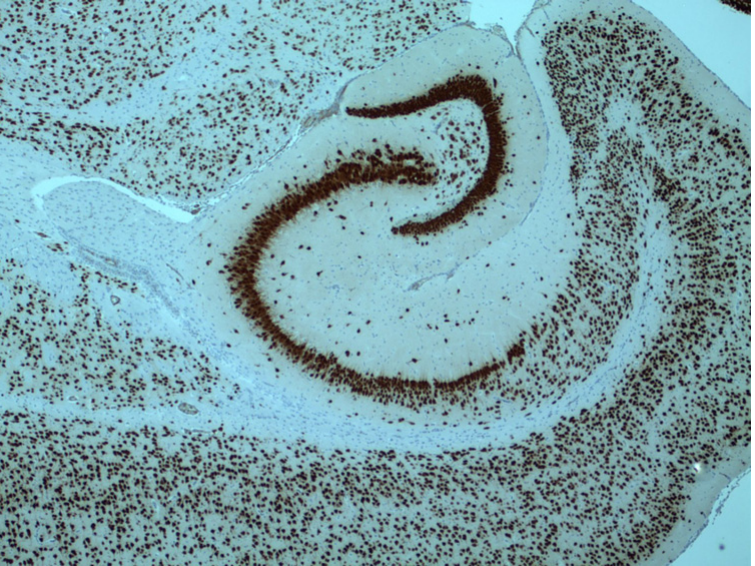
NeuN immunohistochemistry of mouse hippocampus. Photomicrograph (× 40) of NeuN immunoreactivity in mouse hippocampus and adjacent structures 24 hr after ipsilateral ICV LPS injection. Note normal density and distribution of neurons without a cellular infiltrate.

Despite the expectation that LPS would produce a febrile response with widespread damage to brain and an acute encephalitis, we observe that ICV LPS does not yield any of these outcomes (Figure 1) [14]. Indeed, others who injected similar amounts of LPS directly into brain parenchyma also do not observe behavioral changes, tissue damage, or acute inflammatory infiltrate in young wild type (wt) mice [14-18]. We pursued this further by stereological counting of hippocampal CA1 pyramidal neurons 24 and 72 hr following ICV LPS and observed no change in neuron number from untreated controls [14]. These data show that, at least over 3 days following ICV LPS, there is no gross structural damage to brain, no detectable adaptive immune response, and no loss of pyramidal neurons from hippocampal sector CA1.

### Neuronal oxidative damage

**Figure 2:**
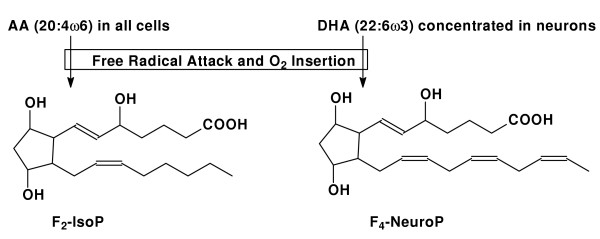
Diagram showing the formation of F_2_-IsoPs and F_4_-NeuroPs.

Numerous methods exist to determine free radical-mediated damage to cells. While most of these function well *in vitro*, important limitations arise in living systems where extensive, highly active enzymatic pathways have evolved to metabolize many of the commonly measured products, such as 4-hydroxynonenal [19]. One method that has been highly replicated as a robust quantitative means of measuring free radical damage *in vivo *is measuring F_2_-isoprostanes (F_2_-IsoPs) [20], products generated from free radical damage to arachidonic acid (AA), that are not extensively metabolized *in situ *(Figure 2). Since AA is present throughout brain and in different cells in brain at roughly equal concentrations, measurement of cerebral F_2_-IsoPs, like all other measures of oxidative damage, reflects damage to brain tissue but not necessarily to neurons. For these reasons, we developed an assay to measure the analogous products generated from DHA, F_4_-NeuroPs [12]. Since DHA is highly concentrated in neuronal membranes, F_4_-NeuroPs offer a unique window into free radical damage to neuronal membranes *in vivo *[21].

We first determined the time course of F_4_-NeuroP accumulation in cerebrum of wt mice exposed to ICV LPS and observed a delayed, transient elevation that peaks at approximately 24 hr after exposure and then returns to baseline by 72 hr post exposure [14]. It is important to note that while detectable neuronal oxidative damage is delayed several hours following ICV LPS, others have shown that altered gene transcription and increased cytokine secretion occur rapidly and peak within a few hours of LPS exposure. As with oxidation of lipoproteins, it is likely that this delay in neuronal oxidative damage is related, at least in part, to the time required to deplete anti-oxidant defenses. Thus, despite the lack of tissue damage, adaptive immune cell infiltrate, or detectable neuron loss, there is significant, reversible free radical damage to neuronal membranes following ICV LPS.

We next used a series of mice, all on the C57Bl/6 genetic background, lacking specific genes to establish the determinants of neuronal oxidative damage in this model. Our results showed that genetic ablation of one co-receptor (CD14), the required adaptor (MyD88), or one arm of the initial signal cascade (the p50 subunit of NF-κB) each completely blocks an LPS-induced increase in cerebral F_4_-NeuroPs (Table 1). Further investigation of mice lacking iNOS, an element of secondary signaling pathways, also completely blocks ICV LPS-induced neuronal oxidative damage. Finally, mice lacking prostaglandin E_2 _receptor subtype 2 (EP2), one of four prostaglandin E_2 _(PGE_2_) receptors expressed in brain and one of the two PGE_2 _receptors expressed by microglia, have no neuronal oxidative damage in response to ICV LPS [16]. There are some important points to consider when interpreting these data. First, not only glia but neurons also will be exposed to LPS in this model. However, we and others have repeatedly shown that primary neurons enriched in cell culture do not respond to LPS [10,11,22-24]; indeed, neurons do not express CD14 and TLR-4 *in vivo *[25,26]. Second, genetic ablation was not specific to cell type. While this limits interpretation of data from some mice, such as p50 -/- and EP2-/- mice because these proteins are expressed by both neurons and glia [27-32], it does not influence interpretation of data from CD14 -/- mice because CD14 expression *in vivo *is restricted to microglia among parencymal cells in brain [25,26]. Thus, these data strongly imply that LPS-activated microglial-mediated paracrine oxidative damage to neurons *in vivo *is dependent on CD14, MyD88, p50 of NF-κB, iNOS, and EP2.

**Table 1:** Neuronal oxidative damage and dendritic degeneration in various knockout mice. Effects of ICV LPS treatment determined at 24 hr in mice homozygous deficient (knockout) for different genes or wildtype (wt) mice all on the C57Bl/6 genetic background (*P < 0.001 by Bonferroni-corrected repeated pair comparisons with ICV saline-exposed mice).

**Knockout**	**Function**	Endpoints*		
		**F_4_-NeuroPs**	**Dendrite Length**	**Spine Density**
None (wt)	N/A	352 + 53*	32 + 4*	37 + 6*
CD14	Receptor	87 + 14	101 + 8	92 + 11
TLR-2	Receptor	----	37 + 5*	51 + 8*
MyD88	Adaptor	98 + 10	96 + 9	102 + 7
p50	Initial Signal Cascade	108 + 11	105 + 7	106 + 10
iNOS	Secondary Signaling	92 + 12	103 + 8	97 + 6
EP2	Secondary Signaling	89 + 9	102 + 12	109 + 5

*% ICV saline-exposed; n > 5 in each group

### Dendritic degeneration

These data left us with an apparent conflict. We have clearly demonstrated neuronal oxidative damage to mouse cerebrum following ICV LPS that is of a magnitude comparable to diseased regions of AD brain [33]. However, there is no apparent structural damage to brain in our study or in others' following ICV or intraparenchymal LPS. We viewed this as a serious potential challenge to the significance of oxidative damage in neurodegeneration. There are differences, of course, between the acute stress of ICV LPS stress and the presumably chronic stress of AD; nevertheless, these data force at least consideration of the question: could oxidative damage to neurons occur *in vivo *to the extent that is observed in AD brain without any neurodegeneration?

**Figure 3:**
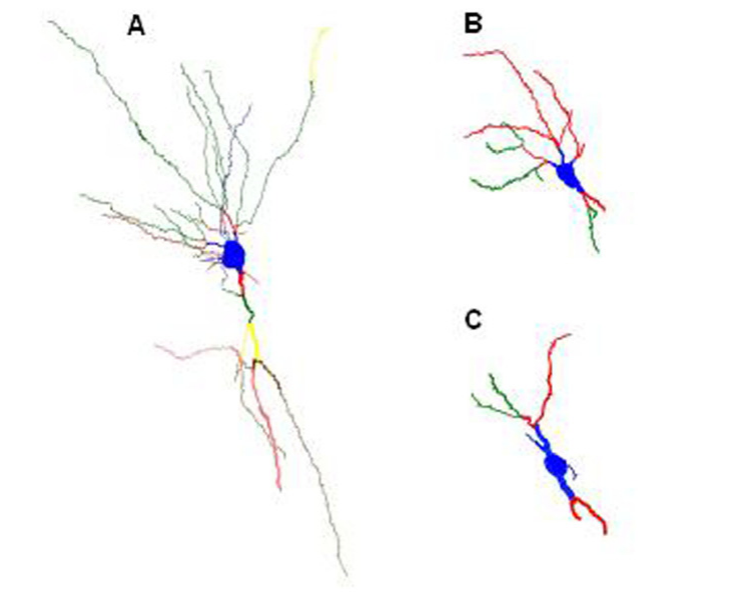
Dendritic degeneration of CA1 pyramidal neurons in mouse hippocampus. Neurolucida renderings of CA1 pyramidal neurons stained by Golgi method; blue is soma and first order dendrites, red is second order dendrites, green is third order dendrites, yellow is fourth order dendrites, brown is fifth order dendrites, and pink is sixth order dendrites. A. Typical pyramidal neuron 24 hr after ipsilateral ICV Saline injection. B and C. Pyramidal neurons following ipsilateral ICV LPS injection showing moderate (B) to severe (C) dendrite shortening and spine loss.

To address this question, we decided to examine directly the dendritic compartment of neurons, which is largely transparent to the standard histological techniques used so far to investigate ICV LPS-induced damage. Using Golgi impregnation and Neurolucida-assisted morphometry of hippocampal CA1 pyramidal neurons [13], we first determined the time course of dendritic structural changes following ICV LPS in wt mice. Our results show a time course similar to neuronal oxidative damage with maximal reduction in both dendrite length and dendritic spine density at approximately 24 hr post LPS and, remarkably, a return to near baseline levels by 72 hr [14] (Figure 3).

**Figure 4:**
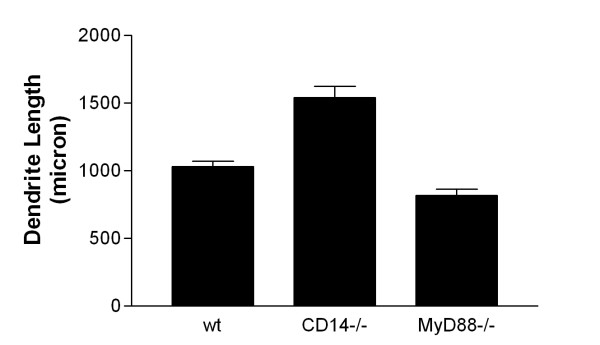
Dendritic arbor in CA1 pyramidal neurons of hippocampus from knockout mice. Adult (6 to 8 week old) wt C57Bl/6, CD14-/-, or MyD88-/- mice received ICV saline 24 hr prior to sacrifice. Tissue sections of hippocampus and surrounding structures were processed for Golgi stain and then evaluated by Neurolucida. Data are dendrite length for CA1 hippocampal pyramidal neurons (n > 15 neurons for each group). One-way ANOVA had P < 0.0001 with Bonferroni-corrected repeated pair comparisons having *P < 0.001 for wt vs. CD14-/- and CD14-/- vs. MyD88-/-.

We next pursued the molecular determinants of ICV LPS-induced dendritic degeneration using the same genetically altered mice that we used above (Table 1). We observed perfect concordance between these results in that lack of a gene that protected cerebrum from neuronal oxidative damage also protected hippocampal CA1 pyramidal neurons from dendritic degeneration and vice versa [14]. Importantly, we had the opportunity to add TLR-2 knockout mice to our analysis. TLR-2, like TLR-4, is one of the plasma membrane TLRs that may be activated by LPS and that also uses CD14 as a co-receptor. Our results show that lack of TLR-2 does not protect hippocampal CA1 pyramidal neurons from ICV LPS-induced neurodegeneration, while lack of CD14 completely protects the dendritic tree of these neurons. Further, it is interesting to note that in mice receiving ICV saline, pyramidal neuron dendrite length (Figure 4), but not spine density, is significantly greater in CD14-/- mice than in wt or MyB88-/- mice, suggesting that even in the absence of specific stimuli like ICV LPS, lack of CD14 perhaps has a net neuroprotective or neurotrophic effect.

### Pharmacologic interventions

**Figure 5:**
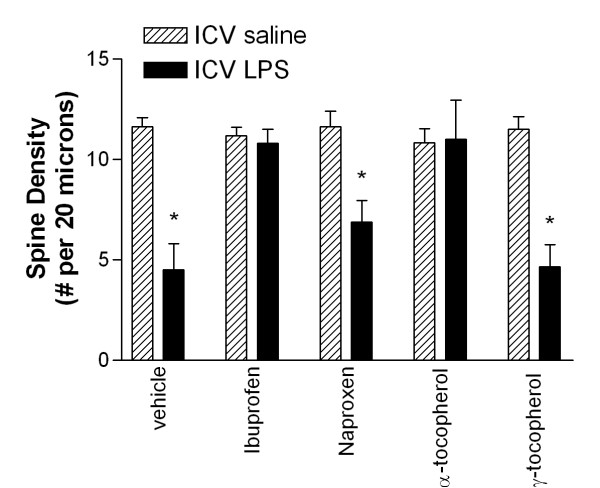
Pharmacologic suppression of dendritic degeneration in CA1 pyramidal neurons of mouse hippocampus. Adult (6 to 8 week old) wt C57Bl/6 mice received ICV saline or ICV LPS 24 hr prior to sacrifice. Tissue sections of hippocampus and surrounding structures were processed for Golgi stain and then evaluated by Neurolucida. Data are dendritic spine density for CA1 hippocampal pyramidal neurons (n > 6 neurons for each group). Two-way ANOVA had P < 0.001 for ICV saline vs. ICV LPS, effect of drugs, and interaction. Post hoc one-way ANOVA showed that no effect of drugs in ICV saline exposed mice. Ibuprofen and α-tocopherol completely protected spine density from ICV LPS exposure (P < 0.01 compared to vehicle treated mice) while naproxen and γ-tocopherol did not significantly protect (P > 0.05).

Considerable controversy surrounds the effective *in vivo *neuroprotective doses of nonsteroidal anti-inflammatory drugs and anti-oxidants that are being evaluated as potenital protectants from AD. Indeed, a major criticism leveled against nonsteroidal anti-inflammatory drugs (NSAIDs) is that the concentrations that appear to be neuroprotective in epidemiologic studies are lower than those that classically considered anti-inflammatory doses. Moreover, there is some data suggesting that some NSAIDs, such as ibuprofen and naproxen, that may differ in their effectiveness as AD protectants despite being equivalent anti-inflammatory agents in peripheral assays of inflammation suggesting alternative mechanisms of action in AD [34]. Therefore, we determined the dose-response relationship for ibuprofen and naproxen in our ICV LPS model utilizing a two-week pre-treatment with each NSAID in drinking water (with concentration expressed as μg/ml drinking water) followed by ICV LPS injection [14]. Neither NSAID alone alters basal levels of cerebral F_4_-NeuroPs. For ibuprofen, the EC_50 _for suppressing ICV LPS-induced F_4_-NeuroPs is between 0.1 and 0.5 μg/ml and the maximal effect is reached by 1.4 μg/ml, considerably lower than the classic anti-inflammatory dose. In contrast, naproxen is without effect up to 1.4 μg/ml and thus an EC_50 _cannot be calculated from these data. As with F_4_-NeuroPs, ibuprofen completely protects both dendrite length and spine density (Figure 5) from the degenerative consequences of ICV LPS; in contrast, naproxen is not significantly protective even at the highest dose. These results are intriguing because some have suggested that ibuprofen may be more effective than naproxen in lowering the risk for AD [34]. The basis for the differing results with these NSAIDs in our experiments are not entirely clear but may derive from pharmacokinetic differences or pharmacodynamic differences in actions other than COX inhibition.

Next, we extended our studies to tocopherols, natural antioxidant products with a number of proposed actions [35] including both anti-oxidant and anti-inflammatory activities [36]. As with NSAIDs, α-tocopherol (AT) or γ-tocopherol (GT) alone does not alter basal F_4_-NeuroP levels or dendritie arbor (not shown). AT partially suppresses ICV LPS-induced F_4_-NeuroPs at 10 mg/kg and completely suppresses F_4_-NeuroP formation and both reduction in dendrite length and reduction in spine density at 100 mg/kg (Figure 5). GT, an isomer of AT that has one-tenth its anti-oxidant activity *in vitro *and lacks a specific transporter in vivo, does not, as expected, protect from neuronal oxidative damage or dendritic degeneration at the same dose.

## Conclusions

Our data show that CD14-dependent activation of cerebral innate immunity leads to an acute, transient increase in oxidative damage to neuronal membranes that coincides with reversible dendritic degeneration. Although we did not directly test TLR-4 deficient mice in our studies, given what is know about LPS receptor activation and the fact that TLR-2-/- mice were not protected from neuronal damage caused by ICV LPS, these data argue strongly for CD14/TLR-4-dependent neuronal damage in our model. Moreover, using a wide array of genetically altered mice, we observed complete concordance between dendritic degeneration and neuronal membrane oxidative damage. In combination, these data suggest that these two events are mechanistically related, perhaps with neuronal membrane oxidative damage being a proximate contributor to dendritic degeneration in the context of innate immune activation.

One obvious, commonly voiced criticism of the model described here is that it produces an acute stress that does not correspond to chronic neurodegenerative diseases. However, it has yet to be shown whether the stress to individual neurons in these protracted diseases truly is chronic or instead the integration of innumerable microscopic acute stresses over many years. Finally, to the extent that CD14-dependent innate immunity activation contributes to neurodegenerative diseases, such as AD and HAD, the model described here provides a convenient means to screen experimental therapeutics and rapidly optimize dosing and timing parameters before moving to more complex animal models or clinical trials.

## List of abbreviations used

AA: arachidonic acid; AD: Alzheimer disease; AT: α-tocopherol; Aβ: amyloid beta; COX-2: cyclooxygenase 2; DHA: docosohexaenoic acid; EP2: prostaglandin E_2 _receptor subtype 2; F_2_-IsoPs: F_2_-isoprostanes; F_4_-NeuroPs: F_4_-neuroprostanes; GT: γ-tocopherol; HAD: HIV-associated dementia; ICV: intracerbroventricular; iNOS: inducible nitric oxide synthase; LPS: lipopolysaccharide; MPO: myeloperoxidase; NSAIDs: nonsteroidal anti-inflammatory drugs; PG: prostaglandin; PGE_2_: prostaglandin E_2_; TLR: Toll-like receptor; wt: wild type.

## Competing Interests

The authors declare that they have no competing interests.
